# Expression of tdTomato and luciferase in a murine lung cancer alters the growth and immune microenvironment of the tumor

**DOI:** 10.1371/journal.pone.0254125

**Published:** 2021-08-19

**Authors:** Lei Huang, Ramireddy Bommireddy, Luis E. Munoz, Rohini N. Guin, Changyong Wei, Amanda Ruggieri, Ashwathi P. Menon, Xiaoxian Li, Mala Shanmugam, Taofeek K. Owonikoko, Suresh S. Ramalingam, Periasamy Selvaraj

**Affiliations:** 1 Department of Pathology and Laboratory Medicine, Emory University School of Medicine, Atlanta, Georgia, United States of America; 2 Department of Hematology and Medical Oncology, Emory University School of Medicine, Atlanta, Georgia, United States of America; National Institute of Child Health and Human Development, UNITED STATES

## Abstract

Imaging techniques based on fluorescence and bioluminescence have been important tools in visualizing tumor progression and studying the effect of drugs and immunotherapies on tumor immune microenvironment in animal models of cancer. However, transgenic expression of foreign proteins may induce immune responses in immunocompetent syngeneic tumor transplant models and augment the efficacy of experimental drugs. In this study, we show that the growth rate of Lewis lung carcinoma (LL/2) tumors was reduced after transduction of tdTomato and luciferase (tdTomato/Luc) compared to the parental cell line. tdTomato/Luc expression by LL/2 cells altered the tumor microenvironment by increasing tumor-infiltrating lymphocytes (TILs) while inhibiting tumor-induced myeloid-derived suppressor cells (MDSCs). Interestingly, tdTomato/Luc expression did not alter the response of LL/2 tumors to anti-PD-1 and anti-CTLA-4 antibodies. These results suggest that the use of tdTomato/Luc-transduced cancer cells to conduct studies in immune competent mice may lead to cell-extrinsic tdTomato/Luc-induced alterations in tumor growth and tumor immune microenvironment that need to be taken into consideration when evaluating the efficacy of anti-cancer drugs and vaccines in immunocompetent animal models.

## Introduction

Fluorescent and luminescent proteins are commonly used for *in vivo* imaging to track cellular processes [1–4]. The use of fluorescence and bioluminescence imaging offers great advantages over traditional methods of tumor measurement using calipers and enables visualization of tumor initiation, progression, metastasis and tumor microenvironment in longitudinal studies [5–9]. The fluorescent dyes frequently used for non-invasive imaging include green fluorescent protein (GFP) and red fluorescent protein (RFP). GFP has an emission peak at 509 nm and is found in jellyfish *Aequorea victoria* [10]. RFP has also been widely used due to its excellent physical-chemical characteristics, such as its brightness and emission spectrum (>620nm) [11, 12]. It is derived from the coral *Discosoma* and named as DsRed, which naturally exists in a tetrameric form [13, 14]. Genetic engineering has created monomeric variants including mCherry, mOrange, mRaspberry, mKO, etc. [14, 15]. In our study, we used tandem dimer Tomato (tdTomato), which is a pseudomonomer that tends to aggregate to form a dimer [14, 15]. Bioluminescence imaging is another popular technique for monitoring tumor growth *in vivo* following transplantation of firefly luciferase-expressing tumor cells into mice [8, 16].

Fluorescence imaging, bioluminescence imaging or a combination of both are frequently used to study different mechanisms of tumor progression and immunotherapy *in vivo* [3, 17–20]. Although the fluorescent and bioluminescent proteins are powerful tools in visualizing tumor microenvironment, transgenic foreign proteins can potentially induce immune response *in vivo* [21–23] and biophotonic emissions may have a detrimental effect on tumor cell function leading to growth inhibition [24–26]. Thus, the use of imaging techniques based on fluorescence and bioluminescence may affect the outcome of intravital studies of anti-cancer therapies, which should be taken into consideration.

In this study, we report that the expression of tdTomato and luciferase (tdTomato/Luc) by a tumor cell line via lentiviral mediated transduction of tdTomato and luciferase encoding gene affects its tumorigenicity and immunogenicity in mice. The lung cancer model that we studied is Lewis Lung Carcinoma (LL/2), which was spontaneously derived from lung carcinoma of C57BL/6 mice by J.S. Bertram in 1951 [27]. Comparison of tdTomato/Luc negative cells with tdTomato/Luc transduced cells revealed that tdTomato/Luc expression by LL/2 cells increased the immunogenicity of tumor cells as evidenced by decreased tumor growth, increased TILs, and decreased G-CSF and MDSC levels.

## Materials and methods

### Cell lines

Lewis Lung Carcinoma (LL/2) derived from spontaneous lung carcinoma of C57BL/6 mice by J.S. Bertram in 1951 [27] was purchased from ATCC (Manassas, VA). LL/2 tumor cells were cultured in Dulbecco’s Modified Eagle Medium (DMEM) (Sigma-Aldrich, St. Louis, MO) supplemented with 10% FBS, 1% HEPES, 1% penicillin and streptomycin, 1% L-glutamine, and 0.1% gentamycin.

### Generation of tdTomato-Luc LL/2 cells

The lentivirus expressing tdTomato and luciferase (tdTomato and luciferase in a pFLUT vector with the expression cassette UB-promoter>Luciferase-T2A-tdTomato) was obtained from the Gene Editing, Transduction and Nanotechnology Core, Skin Biology & Disease Resource-Based Center, Northwestern University, Chicago, IL. LL/2 cells were spinfected by adding virus solution followed by centrifugation to transduce cells as described [28]. Briefly, 1x10^5^ LL/2 cells were incubated with 5 μl of concentrated virus and 5 μg/ml Polybrene Infection/Transfection Reagent (Sigma-Aldrich #TR-1003-G). Then red fluorescence positive cells were sorted using flow cytometry. LL/2 cells co-expressing tdTomato and luciferase are hereafter designated LL/2-tdTomato/Luc cells. LL/2-tdTomato/Luc tumor cells were cultured in Dulbecco’s Modified Eagle Medium (DMEM) (Sigma-Aldrich, St. Louis, MO) supplemented with 10% FBS, 1% HEPES, 1% penicillin and streptomycin, 1% L-glutamine, and 0.1% gentamycin.

### Antibodies

Mouse anti-mouse CTLA-4 mAb (clone 9D9), rat anti-mouse PD-1 mAb (clone RMP1-14), Anti-CD4 (clone GK1.5), anti-CD8 (clone YTS 169.4), and anti-NK1.1 (clone PK136) antibodies were purchased from BioXCell (West Lebanon, NH).

Fluorochrome-conjugated antibodies to CD45 (clone 30-F11), CD3 (clone 17A2), CD4 (clone GK1.5), CD8 (clone 53–6.7), CD19 (clone 1D3), Ly-6G (clone 1A8), CD11b (clone M1/70), CD24 (clone M1/69), CD44 (clone IM7), CD47 (clone MIAP301), PD-L1 (clone 10F.9G2), CD49d (clone R1-2), ICAM-1 (clone YN1/1.7.4), CD80 (clone 16-10A1), CD86 (clone PO3), MHC I (clone 34-1-2S), and MHC II (clone M5/114.15.2) were purchased from BioLegend (San Diego, CA).

### Cell proliferation assay

To measure cell proliferation *in vitro*, LL/2 and LL/2-tdTomato/Luc cells were harvested and resuspended in complete DMEM. Then 1 × 10^5^ cells were plated in a T-25 flask on day 0. The cells were cultured in a 5% CO_2_ incubator at 37°C for three days and were harvested for counting by using trypan blue to determine the cell count and cell viability using a hemocytometer. Then all the cells were transferred to a T-75 flask for continued culturing for another three days. On day 6, the cells were harvested and counted again.

### IFN-γ treatment *in vitro*

LL/2 or LL/2-tdTomato/Luc cells (5 × 10^5^) were treated with 100 ng/ml of recombinant mouse IFN-**γ** (BioLegend Inc., San Diego, CA, USA) in 3 ml complete DMEM. The cells were incubated in a 5% CO_2_ incubator at 37°C. After 72 hours of incubation, cells were harvested for flow cytometry analysis.

### Luminescence assay

To determine the presence of luciferase in the LL/2-tdTomato/Luc cells, Britelite Plus ultra-high sensitivity luminescence reporter gene assay kit (PerkinElmer, Waltham, MA) was used. In a 96-well plate, 5 x 10^4^ cells in 100 μL of phenol red-free DMEM were plated with 100 μL of Britelite Plus reagent in each well. The plate was incubated for 3 minutes for complete cell lysis and full signal generation. Luminescence was measured using BioTek plate reader. Data presented as relative luminescence units (RLU). LL/2 cells used as negative control.

### Flow cytometry

Cell surface markers were analyzed by flow cytometry. Briefly, cells collected from mice were pre-incubated with Fc receptor blocking antibody (BioLegend, TruStain FcX^™^ (anti-mouse CD16/32, clone 93) in FACS buffer (PBS with 2% BCS, 5 mM EDTA, sodium azide 0.05%) at room temperature for 10 minutes to block nonspecific binding of antibodies to immune cells. Cells harvested directly from *in vitro* culture were stained with antibodies without Fc block step. Then, cells were incubated with fluorochrome-conjugated antibodies for 30 minutes with shaking at 4°C. After that, the cells were washed three times with FACS buffer. Then the samples were fixed in 2% formaldehyde with PBS for later analysis or resuspended in FACS buffer for immediate analysis using a FACSCalibur or BD LSRII flow cytometer. Finally, data were analyzed using FlowJo software (Becton, Dickinson and Co., Ashland, OR, USA).

### Tumor model and animals

C57BL/6 mice of 6–8 weeks old were purchased from The Jackson Laboratories (Bar Harbor, ME, USA) and maintained in accordance with guidelines and protocols approved by the Institutional Animal Care and Use Committee (IACUC) of Emory University. For growing LL/2 and LL/2-tdTomato/Luc tumors in C57BL/6 mice, 5 × 10^5^ cells were injected subcutaneously (s.c.) in the flank of the mice. Mice were monitored for tumor growth every three days. Tumor size (mm^2^) was measured using Vernier calipers. Mice were euthanized at the IACUC end point when the longest dimension of the tumor reached 20 mm, usually about 30 days after tumor challenge.

### Immunotherapy and tumor challenge studies

In the ICI immunotherapy study, four doses of anti-PD-1 (Clone RMP1-14, Bio X Cell) and anti-CTLA-4 antibodies (Clone 9D9, Bio X Cell) were injected intraperitoneally (200 μg/ dose) every four days starting day 4 after tumor cell challenge. Mice were monitored for tumor growth every three days. At the IACUC end point, mice were euthanized to harvest organs for analysis.

### *In vivo* cellular depletion

CD4^+^ T cells, CD8^+^ T cells, and NK cells were depleted by intraperitoneal (i.p.) injection of 200 μg of anti-CD4 (clone GK1.5), anti-CD8 (clone YTS 169.4), and anti-NK1.1 (clone PK136) antibodies respectively in antibody dilution buffer pH 7.0 (Bio X Cell). Cellular depletion was confirmed by flow cytometry analysis of CD4^+^ T cells, CD8^+^ T cells and NK cells in the blood samples of mice within 24 hours after the first depletion antibody injection. The next day, mice were challenged with 5 × 10^5^ LL/2-tdTomato/Luc cells and another four doses of cellular depletion antibodies were given every four to five days post tumor challenge to deplete newly formed lymphocytes.

### Cell preparation from tumor and spleen tissues

Tumor tissue from euthanized mice was minced using curved scissors or scalpels. Then the minced tumor tissue was transferred to a 15 ml conical tube and incubated in 2 ml of freshly prepared complete DMEM containing 1 mg/ml collagenase type IV (Sigma-Aldrich Co. St Louis, MO), 10% DNase (Invitrogen Inc., Waltham, MA) and 10% Liberase (Roche Inc., Basel, Switzerland) in a shaking incubator at 37°C for 30–40 minutes. After digestion, single cell suspensions were prepared by passage through a cell strainer. The tumor digest was centrifuged, and the cell pellet was resuspended in PBS. Then the cells were stained with fluorochrome-conjugated antibodies after Fc block for flow cytometry analysis as described above.

To prepare splenocytes, the spleens were mechanically disrupted using the back of a syringe plunger to pass through a cell strainer directly after tissue harvesting. Then the splenocytes were incubated with red blood cell (RBC) lysis buffer (Sigma-Aldrich, St. Louis, MO) (5ml/spleen) at room temperature for 5 minutes with occasional shaking for lysing the RBCs. The cells were washed with PBS, counted, and stained for flow cytometry analysis.

### Cytokine assays

The mice were anesthetized with isoflurane in an induction chamber. The facial vein was punctured with an animal lancet (5 mm) and the blood samples were collected in a collection tube with Acid Citrate Dextrose (ACD) solution (Sigma-Aldrich, St. Louis, MO). The blood samples were then centrifuged to collect plasma. Cytokines present in circulation were detected in diluted plasma (1:10) by standard sandwich ELISA (BioLegend Inc., San Diego, CA, USA).

For measuring cytokines in cell culture supernatants, LL/2 or LL/2-tdTomato/Luc cells were plated at a concentration of 1 x 10^6^ cells/4 ml/well in a six-well tissue culture plate. The plate was incubated in a 5% CO_2_ incubator at 37°C for 48 hours. After incubation, cell culture supernatants were harvested and analyzed for cytokines by sandwich ELISA. Specifically, we determined the presence of TNF-α, IL-6, GM-CSF, G-CSF, VEGF, and IFN-**γ**.

### Enzyme-Linked Immunosorbent Assay (ELISA)

To determine the cytokine levels in the plasma and cell culture supernatants, a standard sandwich ELISA was performed. Briefly, 96-well ELISA plates were coated with capture antibody in coating buffer and incubated at 4°C overnight. Then the plates were washed three times with wash buffer (PBS-0.05% Tween). After blocking for 2 hours with 1% BSA in PBS at room temperature, the plates were washed again three times with wash buffer. Then the plates were incubated with the samples and standards for 2 hours at room temperature, followed by three times of washing. The plates were incubated with biotinylated detection antibody for 1 hour at room temperature, followed by another three times of washing. Next, the plates were incubated with streptavidin-peroxidase for 30 minutes at room temperature and washed five times with washing buffer. Then, the plates were incubated with TMB substrate for approximately 10 minutes at room temperature. The reaction was stopped with 2 M H_2_SO_4_ and the plates were read in a BioTek plate reader at 450 nm.

### Statistical analysis

All the statistical analyses and graphs were performed using GraphPad Prism software. Significance of mean differences was determined by using unpaired student t-tests. Values of p<0.05 were considered significant (*p < 0.05, **p < 0.01, ***p ≤ 0.001). Survival curves were plotted and analyzed using Kaplan-Meier analysis.

## Results

### TdTomato/Luc expression does not alter cell surface markers of LL/2 cells

**Fig 1:**
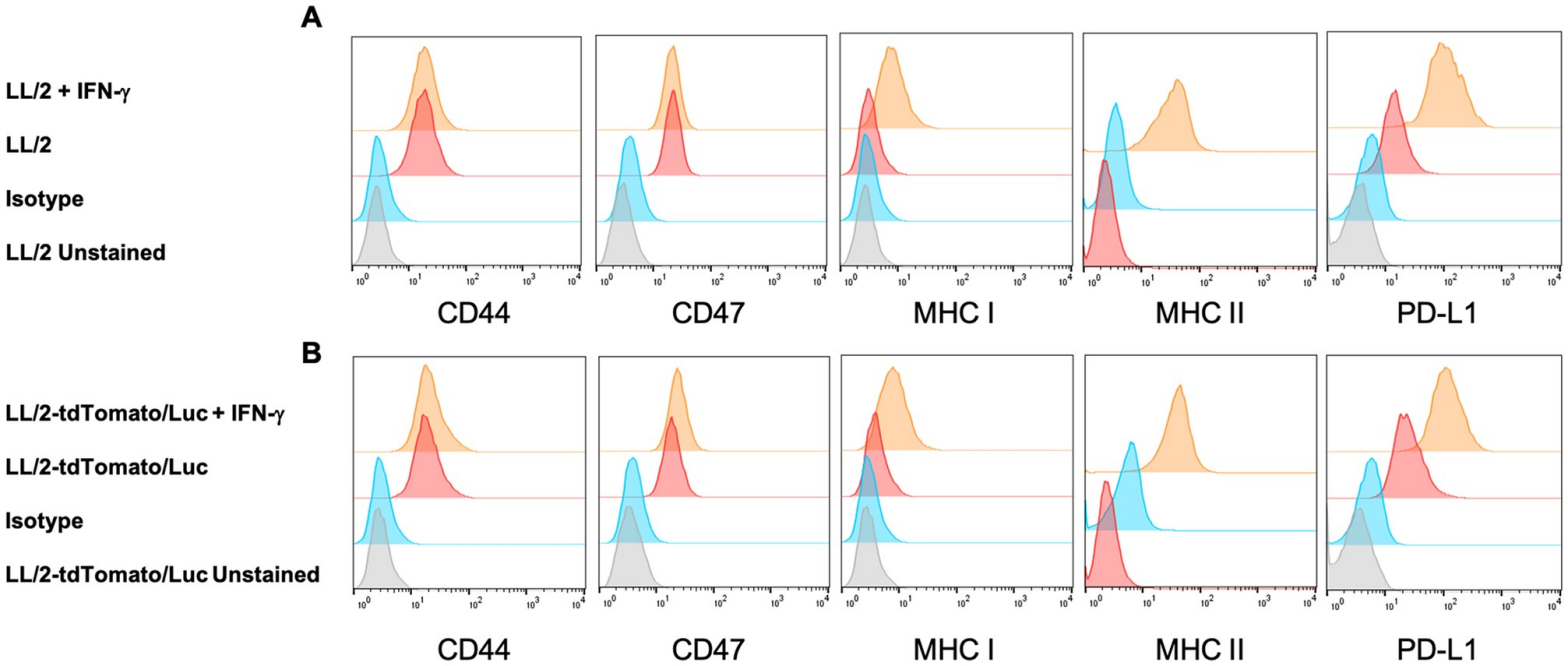
TdTomato/Luc expression does not alter cell surface markers of LL/2 cells. Flow cytometry plots of surface markers expressed on LL/2 and LL/2-tdTomato/Luc cells with or without IFN-**γ** treatment. LL/2 cells (A) and LL/2-tdTomato/Luc cells (B) were cultured with or without IFN-**γ** (100 ng/ml) for 72 hours. Cells were harvested and stained for FACS analysis as described in the Methods section.

We first confirmed the expression of tdTomato and luciferase in LL/2-tdTomato/Luc cells by flow cytometry and luciferase assays respectively (S1A and S1B Fig). To investigate whether the expression of tdTomato/Luc by LL/2 cells alters the expression of cell surface markers, we analyzed the cells by flow cytometry. The specific markers that we analyzed include tumor stem cell markers (CD24, CD44), markers of immunosuppression (CD47, PD-L1), markers of cell adhesion (CD49d, ICAM-1), markers of lymphocytes co-stimulation (CD80, CD86), and markers of antigen presentation (MHC class I, MHC class II). Among these markers, both cell lines express CD44, CD47, and PD-L1 but not the other markers we analyzed (Fig 1A and 1B and S2A and S2B Fig). This demonstrates that these two cell lines are comparable in the selected markers except for the tdTomato/Luc expression.

Since tumors are exposed to IFN-**γ** secreted by tumor-infiltrating immune cells under *in vivo* conditions, we treated LL/2 and LL/2-tdTomato/Luc cells with IFN-**γ** to assess the potential changes in the upregulation of IFN-**γ**-inducible cell surface markers. We found that IFN-**γ** treatment upregulated the levels of MHC class I, MHC class II, and PD-L1 in both types of cells to the same degree but did not affect the expression of other markers (Fig 1A and 1B and S2 Fig). Therefore, except for tdTomato/Luc expression, LL/2 and LL/2-tdTomato/Luc cells have the same phenotype with or without IFN-**γ** treatment.

### TdTomato/Luc expression retards the growth of LL/2 cells *in vivo*

**Fig 2:**
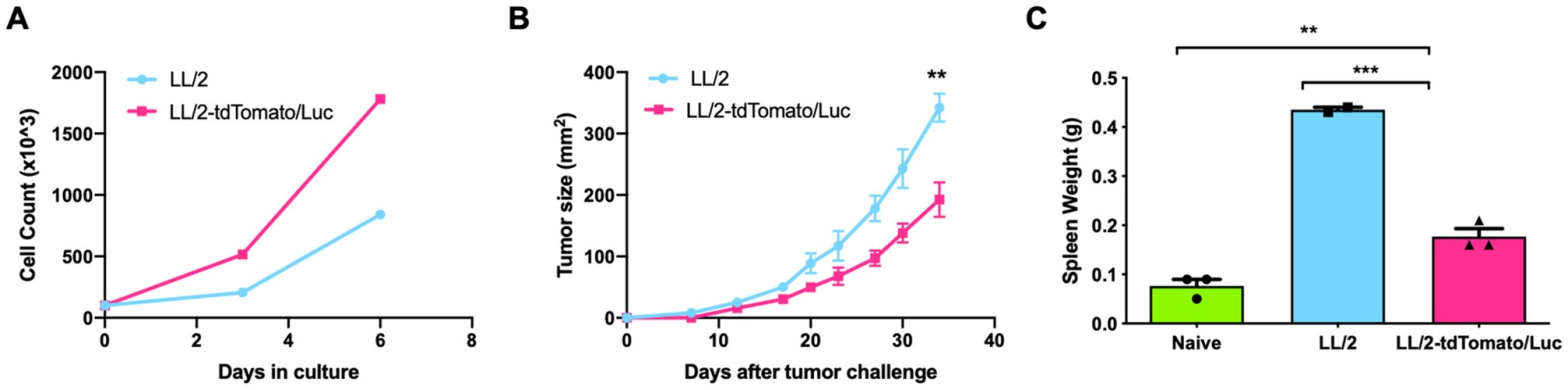
TdTomato/Luc expression by LL/2 cells inhibits its tumorigenicity. (A) Tumor cell growth *in vitro*, and (B) tumor growth *in vivo*. (C) Spleen weight of tumor-bearing mice (n = 3). Mean ± SEM is plotted. Significance of differences was determined by using unpaired student t-tests. Values of p<0.05 were considered significant (*p < 0.05, **p < 0.01, ***p ≤ 0.001).

To investigate the effect of tdTomato/Luc expression on the kinetics of LL/2 cell proliferation, we assessed the cell growth potential of the two cell lines *in vitro*. LL/2 and LL/2-tdTomato/Luc cells were plated in a T-25/T-75 flasks at the same seeding density (1 × 10^5^/5 ml media) and were counted 3 and 6 days later. Our data show that LL/2-tdTomato/Luc cells grow at a faster rate (about two-fold increase) compared to LL/2 cells *in vitro* (Fig 2A).

Next, the effect of tdTomato/Luc expression on LL/2 tumorigenicity was analyzed. C57BL/6 mice were challenged with 5 × 10^5^ cells s.c. in the flank and the tumor growth was monitored. We observed that LL/2-tdTomato/Luc tumors grew slower than LL/2 tumors *in vivo* (Fig 2B). Since splenomegaly due to accumulation of immunosuppressive MDSCs was observed in this model, we also compared the spleen weight between both groups. We found that the spleen weight of LL/2-tdTomato/Luc-tumor bearing mice was significantly smaller than that of LL/2-tumor bearing mice (Fig 2C). Therefore, these results suggest that tdTomato/Luc-expressing LL/2 cells exhibit reduced growth/tumorigenicity *in vivo*, which may be related to a reduced accumulation of MDSCs in the spleen.

### TdTomato/Luc expression by LL/2 cells increases immunogenicity and inhibits tumor induced G-CSF and MDSC levels

**Fig 3:**
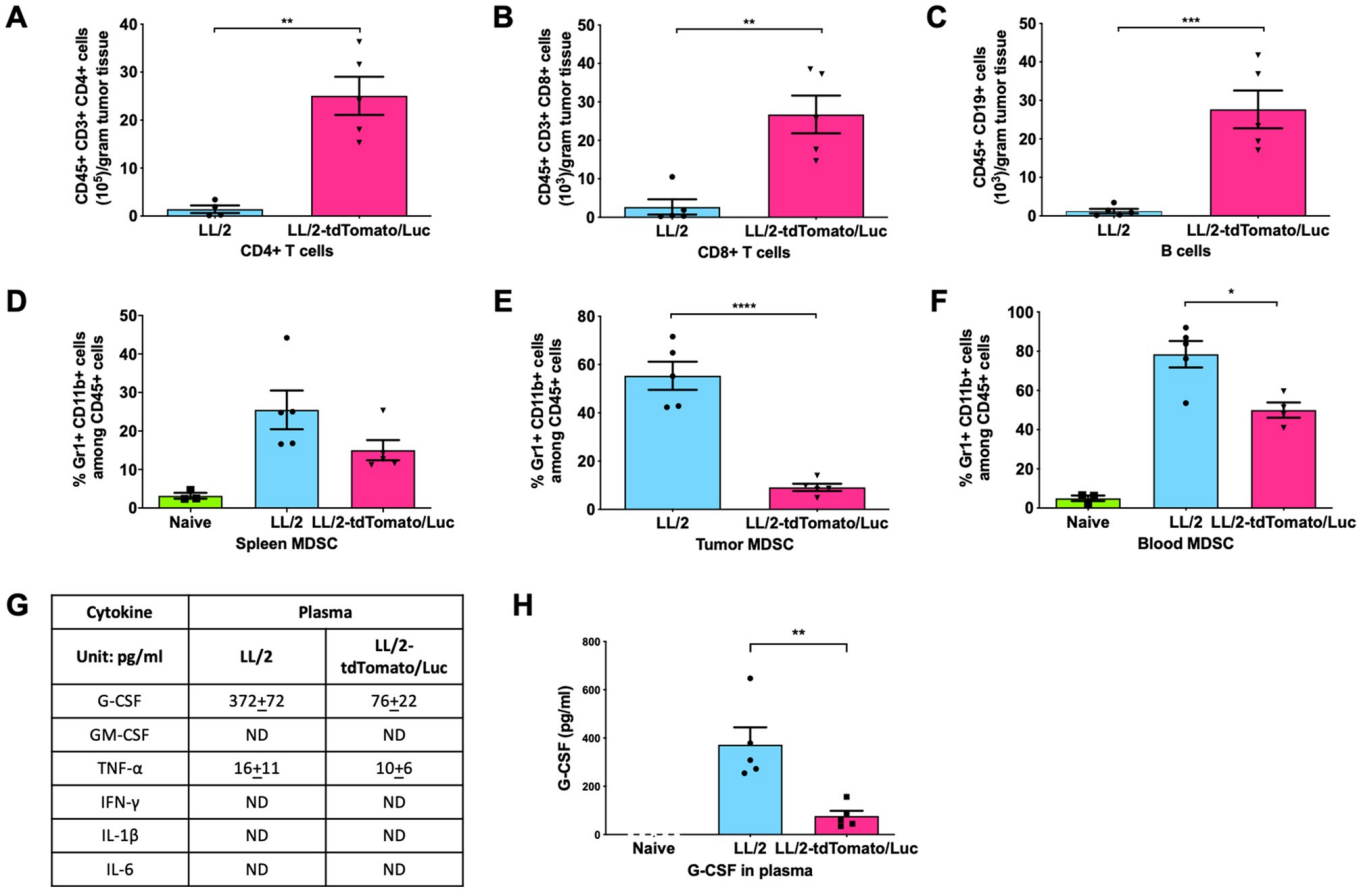
TdTomato/Luc expression by LL/2 cells increases tumor immunogenicity and inhibits tumor-induced G-CSF and MDSC levels. (A-C) Tumors from Fig 2B were harvested and analyzed for tumor infiltrating cell counts of (A) CD4+ T cells (CD45+, CD3+, CD4+), (B) CD8+ T cells (CD45+, CD3+, CD8+), and (C) B cells (CD45+, CD19+) are shown. (D-F) MDSC levels in the spleen, tumor and blood samples of LL/2 and LL/2-tdTomato/Luc tumor bearing mice. (G) The levels of common MDSC-inducing factors in the plasma of mice including G-CSF, GM-CSF, TNF-α, IFN-**γ**, IL-1β, and IL-6. (H) The concentration of G-CSF in the plasma of mice. Mean ± SEM is plotted. Significance of differences was determined by using unpaired student t-test. Values of p<0.05 were considered significant (*p < 0.05, **p < 0.01, ***p ≤ 0.001).

To test the hypothesis that the decreased growth of LL/2-tdTomato/Luc cells *in vivo* is due to an altered tumor immune microenvironment in LL/2-tdTomato/Luc tumors, tumor-infiltrating immune cells in the tumors from Fig 2B harvested at the IACUC endpoint were analyzed by flow cytometry. We found that there were significantly higher numbers of CD4^+^ T cells (CD45^+^, CD3^+^, CD4^+^), CD8^+^ T cells (CD45^+^, CD3^+^, CD8^+^), and B cells (CD45^+^, CD3^-^, CD19^+^) in LL/2-tdTomato/Luc tumors compared to LL/2 tumors (Fig 3A–3C). Since MDSCs are precursor myeloid cells with immunosuppressive and tumor-promoting capabilities [29], we also assessed the level of MDSCs (Gr-1^+^ CD11b^+^) in blood, spleen and tumor samples by flow cytometry analysis. Compared to the LL/2-tumor bearing mice, LL/2-tdTomato/Luc-tumor bearing mice have a significantly decreased levels of MDSCs in the spleen, tumor and blood (Fig 3D–3F).

Furthermore, we analyzed the cytokine levels in plasma to determine whether LL/2 tumors secrete more MDSC-inducing/activating factors than LL/2-tdTomato/Luc tumors *in vivo*. Numerous studies have demonstrated that the immunosuppressive function of MDSC is upregulated by inflammatory molecules such as G-CSF, GM-CSF, VEGF, IL-6, IL-1β, TNF-α, COX-2, IFN-γ, etc. [30]. Among these MDSC-inducing/activating factors, we have analyzed the secretion of cytokines G-CSF, GM-CSF, TNF-α, IL-6, IL-1β and IFN-γ in plasma by ELISA. Our data show that the level of cytokines GM-CSF, TNF-α, IL-6, IL-1β and IFN-γ was not detectable, so they are not likely contributing to the increase in MDSCs (Fig 3G). However, we observed that the level of G-CSF was significantly decreased in the plasma of LL/2-tdTomato/Luc-tumor bearing mice compared to LL/2-tumor bearing mice (Fig 3H). To determine whether the decreased G-CSF level in plasma is due to reduced secretion by LL/2-tdTomato/Luc-tumor cells, we assessed the level of G-CSF in the culture supernatants of LL/2 and LL/2-tdTomato/Luc cells *in vitro*. G-CSF levels were below detection levels in the supernatants collected from LL/2 and LL/2-tdTomato/Luc cell cultures. Therefore, these data suggest that LL/2 tumor cells do not secrete G-CSF directly, suggesting indirect mechanism of G-CSF production induced by tumors, which contribute to an increase in circulating G-CSF and consequently an increased level of MDSCs.

### CD4^+^ T cells, CD8^+^ T cells, and NK cells play a role in inhibiting LL/2-tdTomato/Luc tumor growth *in vivo*

**Fig 4:**
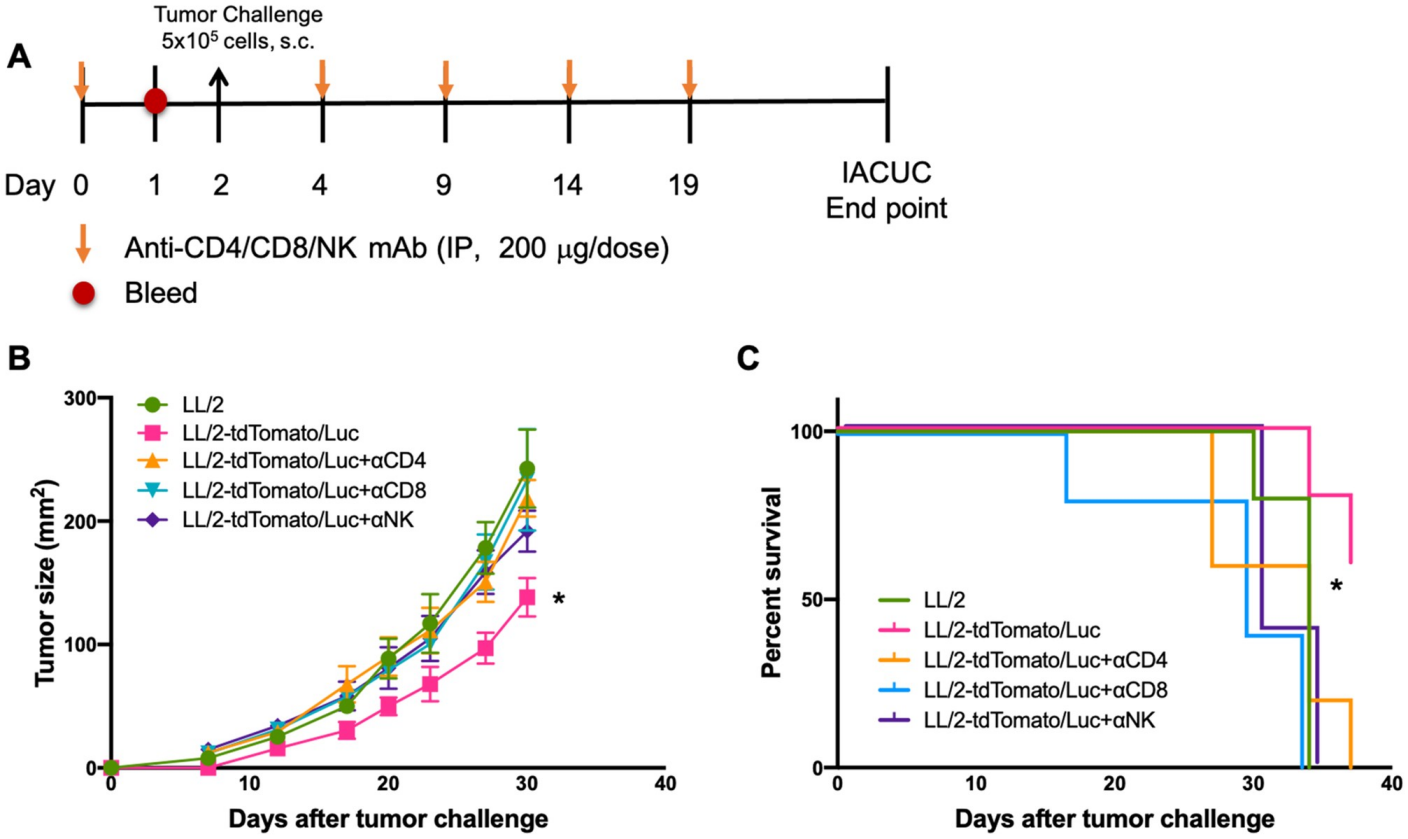
LL/2-tdTomato/Luc tumor growth is inhibited by CD4+ T cells, CD8+ T cells and NK cells *in vivo*. (A) Experimental design of *in vivo* cell depletion study (n = 5). (B) Tumor growth and (C) tumor-free survival. Data were analyzed by unpaired student t-tests on day 30. Mean ± SEM is plotted. Values of p<0.05 were considered significant. *p < 0.05 LL/2-tdTomato/Luc vs. all the other groups. Tumor-free survival was analyzed using Kaplan-Meier analysis. Values of p<0.05 were considered significant. *p < 0.05 LL/2-tdTomato/Luc vs. all the other groups.

To further investigate the types of immune cells that retarded the growth of LL/2-tdTomato/Luc cells in mice, an *in vivo* immune cell depletion study was conducted (Fig 4A). Compared to the control mice challenged with LL/2-tdTomato/Luc tumor cells, depletion of CD4^+^ T cells, CD8^+^ T cells, or NK cells resulted in elevated tumor growth comparable to LL/2 cell challenge group and led to significant reduction in survival of the mice (Fig 4B and 4C). This suggests that CD4^+^ T cells, CD8^+^ T cells, and NK cells all play a role in inhibiting LL/2-tdTomato/Luc tumor growth *in vivo*.

### ICI blockade does not inhibit LL/2 or LL/2-tdTomato/Luc tumor growth

**Fig 5:**
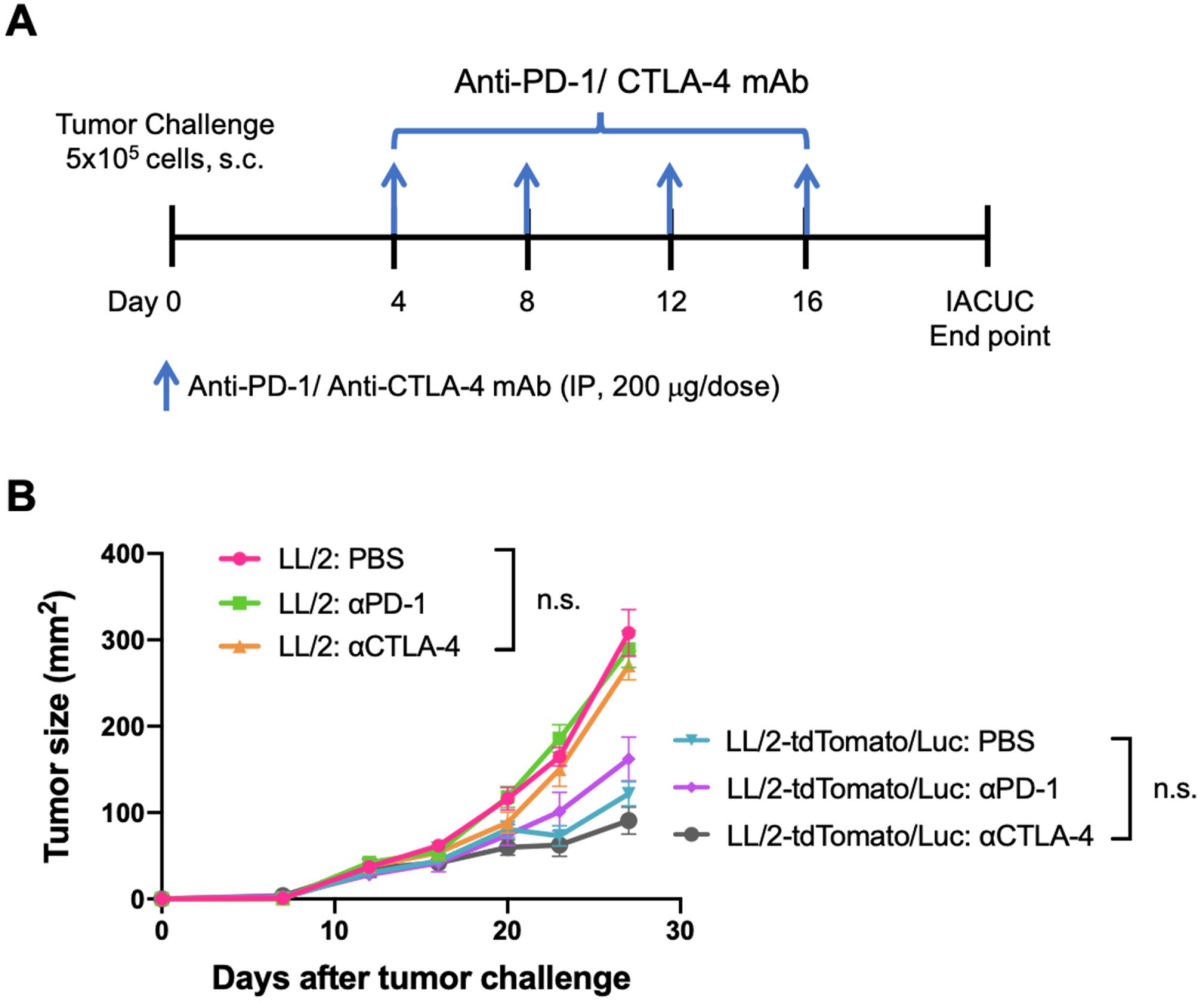
ICI blockade does not inhibit LL/2 or LL/2-tdTomato/Luc tumor growth. (A) Experimental design of the *in vivo* study (n = 5). (B) Tumor size of mice treated with anti-PD-1 or anti-CTLA-4 mAb. Mean ± SEM is plotted. Significance of differences was determined by using unpaired student t-test. Values of p<0.05 were considered significant.

To investigate whether the expression of transgenic proteins affect the efficacy of immune checkpoint inhibitors (ICI) therapies in LL/2 model, we determined the effect of anti-PD-1 mAb and anti-CTLA-4 mAb on the growth of LL/2 and LL/2-tdTomato/Luc tumors. C57BL/6 mice were inoculated with 5 x 10^5^ tumor cells s.c. on day 0 and administered with 4 doses of anti-PD-1 or anti-CTLA-4 mAb (200 μg) every four days starting on day 4 (Fig 5A). In both LL/2 and LL/2-tdTomato/Luc tumor models, neither anti-PD-1 nor anti-CTLA-4 mAb reduced the tumor size although mice challenged with LL/2-tdTomato/Luc cells had significantly smaller tumors compared to mice challenged with LL/2 cells (Fig 5B). This further suggests that LL/2 tumor model is ICI resistant and tdTomato/Luc expression does not alter its response to ICI.

## Discussion

Proteins used for tumor imaging such as luciferase, GFP and RFP are frequently used as selectable markers for transgene expression and have various applications in tracking biological processes. Live animal imaging based on fluorescence or bioluminescence signals has become a popular technique for tracking tumor growth and studying the mechanisms of cancer therapies [1, 5, 7, 8]. TdTomato has been frequently used as a substitute for GFP for cell labeling due to some advantages such as stable expression and no need of tissue fixation [31–33]. Numerous studies have documented immunogenicity of GFP, but only few examined immune response against RFP [34–38]. Moreover, the effect of luciferase expression on tumor growth is controversial [24, 39]. Some findings suggest build-up of oxyluciferin, the product of luciferase-luciferin reaction, may cause oxidative damage to the cells resulting in inhibition of tumor growth while others show that luciferase and its biophotonic activity were not sufficient to cause the detrimental effect [39–41].

In this study, we transfected murine LL/2 lung cancer cell line with tdTomato/Luc-expressing lentivirus and analyzed whether tdTomato/Luc expression by LL/2 cells influenced their tumorigenicity and immunogenicity in mice. In addition to tumor growth, we also compared the spleen weight between two groups since splenomegaly is associated with immunosuppressive MDSCs in many tumors [42, 43]. Our data show that tdTomato/Luc expression by LL/2 cells decreased tumor growth and reduced splenomegaly in mice. Others have reported that fluorescent proteins induce oxidative stress in cells, leading to cell death [44, 45] and luciferase-luciferin reaction may also cause oxidative damage to the cells [24, 41]. However, since LL/2-tdTomato/Luc cells proliferated at a faster rate *in vitro*, the decreased growth of LL/2-tdTomato/Luc cells *in vivo* is likely attributed to the tumor extrinsic mechanisms. Further, comparison of surface antigens on LL/2 and LL/2-tdTomato/Luc cell lines showed that lentiviral transduction of LL/2 cell line with tdTomato/Luc does not alter the expression of cell surface markers with or without IFN-γ treatment. Since IFN-γ plays a critical role in regulating immunomodulatory molecules such as PD-L1, MHC class I and class II in the TME, the similar expression levels of cell surface markers on both cell lines after IFN-γ treatment suggests that tdTomato/Luc transduction did not impact cell surface immunomodulatory molecules.

Our study shows LL/2-tdTomato/Luc-tumor bearing mice have a smaller tumor size, a higher level of TILs, and lower levels of G-CSF and MDSCs in blood in comparison to LL/2-tumor bearing mice. The mechanism behind the reduced level of G-CSF in the LL/2-tdTomato/Luc-tumor bearing mice is not clear. One possible mechanism in tdTomato/Luc-mediated anti-tumor effect may be that the presentation of tdTomato/Luc antigen in tumor cells primes and activates immune cells in the TME leading to decreased levels of immunosuppressive G-CSF and MDSC. This, in turn, allows more infiltration of T lymphocytes into tumor tissue, which control the tumor growth. The involvement of immune cells in controlling the growth of LL/2-tdTomato/Luc cells is further confirmed by the lymphocyte depletion studies, which showed that the LL/2-tdTomato/Luc tumor growth in mice depleted of CD4, CD8 or NK cells is similar to the LL/2 tumor growth.

Our data show that LL/2 subcutaneous tumor growth is resistant to anti-PD-1 and anti-CTLA-4 antibody therapies. In spite of reduced tumor growth, the LL/2-tdTomato/Luc cells remained resistant to ICI blockade similar to the parental cell line. Our findings are in agreement with a previous study implicating that the mice implanted with luciferase-expressing tumors may develop an immune response to the CTL epitope of luciferase resulting in reduced tumor growth and metastatic activity [26]. However, one of the limitations of our study is that it does not show whether the enhancement of immunogenicity in mice is due to the expression of tdTomato, luciferase or both. Therefore, further studies are needed to investigate the contribution of individual proteins for tumor immunogenicity.

Overall, our study shows that tdTomato/Luc expression by LL/2 cells changes the tumor microenvironment and alters the anti-tumor immunity. This indicates that using tdTomato/Luc as a selectable marker may augment the anti-tumor responses induced by therapeutic agents, which potentially confounds the interpretation of experimental data. Therefore, these factors need to be taken into consideration when interpreting data using fluorescent and bioluminescent imaging for *in vivo* visualization of tumors in investigational studies.

## Supporting information

S1 Fig LL/2-RFP/Luc cells express RFP and luciferase. LL/2 and LL/2-RFP/Luc cells were analyzed for RFP expression (PE-CF594) by flow cytometry (A) and luciferase expression by luminescence assay (B). LL/2 cells were used as negative control for the RFP and luciferase assays. Assays were repeated at least three times.
(TIF)

S2 Fig TdTomato/Luc expression does not alter cell surface markers of LL/2 cells. Flow cytometry plots of surface markers expressed on LL/2 and LL/2-tdTomato/Luc cells with or without IFN-γ treatment. LL/2 cells (A) and LL/2-tdTomato/Luc cells (B) were cultured with or without IFN-γ (100 ng/ml) for 72 hours. Cells were harvested and stained for FACS analysis as described in the Methods section.
(TIF)
