# Supplementary figures and images for: Expression of tdTomato and luciferase in a murine lung cancer alters the growth and immune microenvironment of the tumor
Source: PLoS One. 2021 Aug 19;16(8):e0254125. doi: 10.1371/journal.pone.0254125 (PMC8376001; doi:10.1371/journal.pone.0254125)

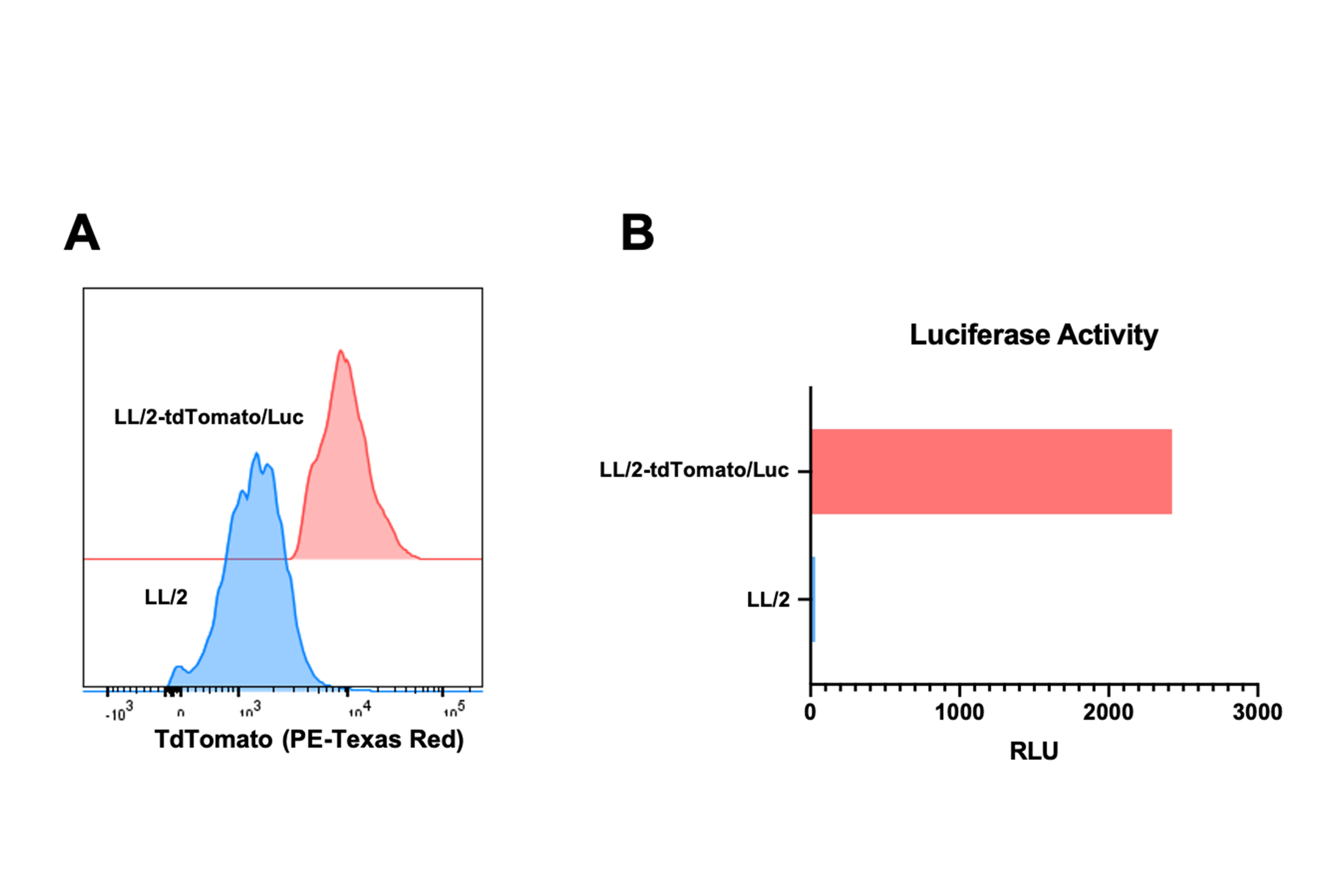

Supplement: S1 Fig — LL/2 and LL/2-RFP/Luc cells were analyzed for RFP expression (PE-CF594) by flow cytometry (A) and luciferase expression by luminescence assay (B). LL/2 cells were used as negative control for the RFP and luciferase assays. Assays were repeated at least three times. (TIF) [file pone.0254125.s001.tif]

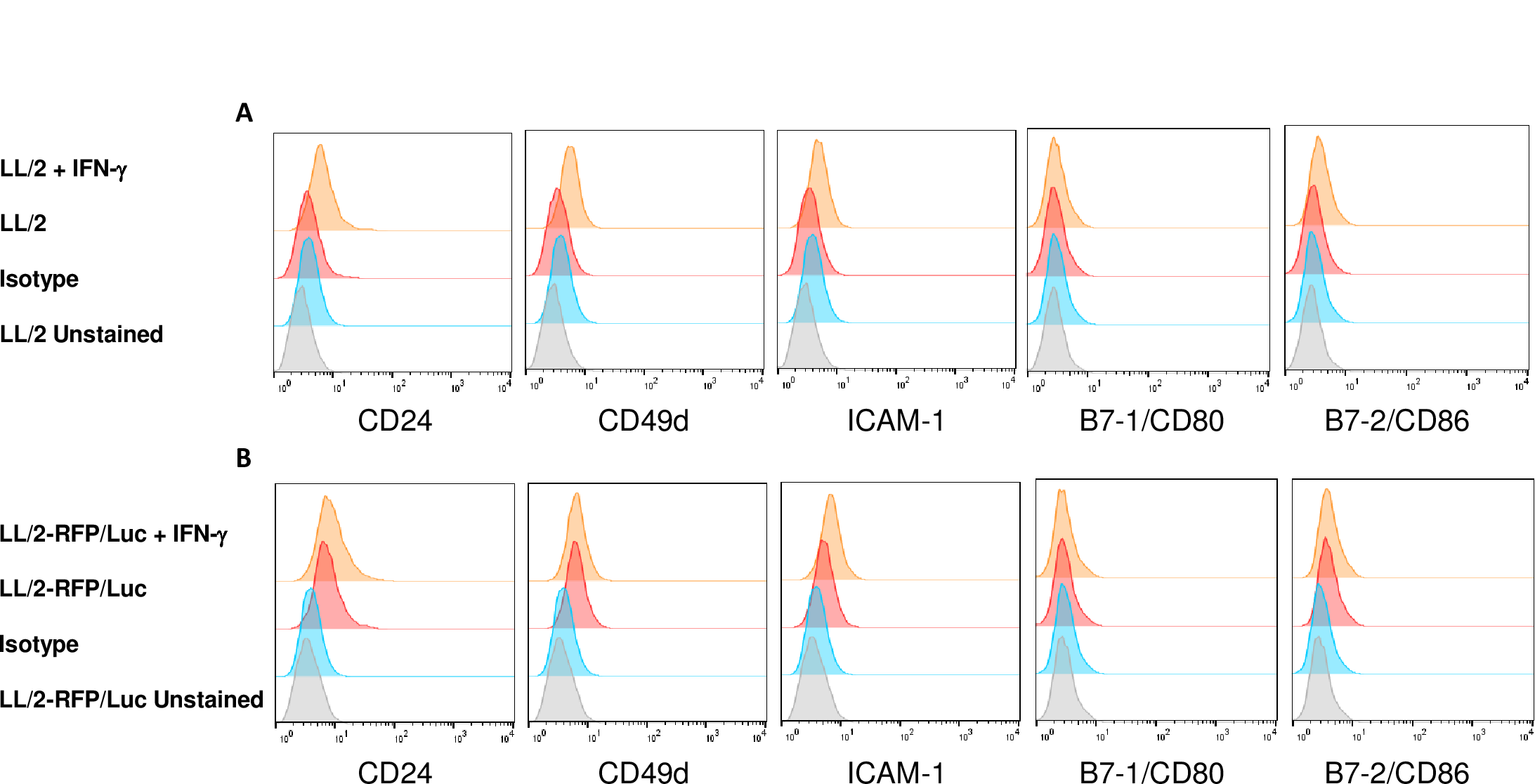

Supplement: S2 Fig — Flow cytometry plots of surface markers expressed on LL/2 and LL/2-tdTomato/Luc cells with or without IFN-γ treatment. LL/2 cells (A) and LL/2-tdTomato/Luc cells (B) were cultured with or without IFN-γ (100 ng/ml) for 72 hours. Cells were harvested and stained for FACS analysis as described in the Methods section. (TIF) [file pone.0254125.s002.tif]
